# The association between weight-adjusted-waist index and total bone mineral density in adolescents: NHANES 2011–2018

**DOI:** 10.3389/fendo.2023.1191501

**Published:** 2023-05-17

**Authors:** Xiaohua Wang, Shuo Yang, Gansheng He, Lin Xie

**Affiliations:** Department of Spine Surgery, Affiliated Hospital of Integrated Traditional Chinese and Western Medicine, Nanjing University of Chinese Medicine, Nanjing, China

**Keywords:** bone mineral density, NHANES, obese, osteoporosis, weight-adjusted-waist index, adolescent

## Abstract

**Introduction:**

The weight-adjusted waist index (WWI) serves as an innovative obesity measure, seemingly surpassing body mass index (BMI) and waist circumference (WC) in evaluating lean and fat mass. This study aimed to explore the relationship between WWI and total bone mineral density (BMD) in US adolescents.

**Methods:**

This population-based study investigated adolescents aged 8–19 years with comprehensive WWI and total BMD data from the National Health and Nutrition Examination Survey (NHANES) 2011–2018. WWI was computed by dividing WC by the square root of body weight. Weighted multivariate linear regression and smoothed curve fitting were employed to examine linear and non-linear associations. Threshold effects were determined using a two-part linear regression model. Additionally, subgroup analyses and interaction tests were conducted.

**Results:**

Multivariate linear regression analysis revealed a significant negative association between WWI and total BMD in 6,923 US adolescents aged 8–19 years [β = -0.03, 95% CI: (-0.03, -0.03)]. This negative correlation remained consistent across all subcategories, with the exception of age, encompassing gender,ethnicity, and diabetes status subgroups. Furthermore, a non-linear relationship and saturation effect between WWI and total BMD were identified, with an inflection point at 9.88 cm/√kg.

**Conclusions:**

Our research demonstrated a notable negative relationship and saturation effect between WWI and total BMD among US adolescents.

## 1 Background

Osteoporosis (OP) is a systemic degenerative bone disorder characterized by diminished bone density and compromised bone microarchitecture, resulting in bone fragility and fractures (1–3). A global epidemiological investigation estimated that roughly 200 million individuals suffer from OP worldwide, with this number increasing annually (4). Childhood and adolescence represent critical phases for bone development, with bone mass typically peaking in late adolescence (5, 6). Numerous studies have shown that bone mineral density (BMD) is traceable from childhood to adolescence and into adulthood (7, 8). Consequently, bone metabolism during childhood and adolescence plays a vital role in preventing OP in later life (9).

Obesity is defined by abnormal or excessive body fat, which negatively impacts health and is closely associated with the development of various chronic diseases (10). Childhood obesity is a major concern in the United States, affecting an estimated 14.4 million children and adolescents (11). While body mass index (BMI) and waist circumference (WC) are the predominant measures of obesity, the obesity paradox has cast doubt on the utility of BMI. It has been proposed that BMI fails to distinguish between lean and fat mass and is subject to influence by factors such as age, gender, and ethnic differences (12, 13). In contrast, WC is considered a superior indicator of body fat for predicting obesity-related diseases compared to BMI, as it demonstrates a strong correlation with abdominal fat accumulation (14, 15). However, the substantial correlation between WC and BMI constrains the utility of WC as an independent obesity marker. Consequently, in 2018, Park et al. proposed a new obesity index, the weight-adjusted waist index (WWI), which standardizes WC and body weight, rendering it easy to measure (16). Furthermore, WWI highlights the advantages of WC and reduces the correlation between WC and BMI by primarily reflecting weight-independent central obesity. Numerous studies have demonstrated a positive relationship between WWI and the onset of hypertension, diabetes, and even all-cause and cardiovascular mortality (17–19).

As ongoing research progresses, scholars observed a potential correlation between body fat composition and bone tissue development. Consequently, tracking this development through easily quantifiable obesity indicators proves valuable. To date, no studies have established a connection between WWI and total BMD. The objective of this investigation was to examine the relationship between WWI and total BMD in US adolescents aged 8–19 years, utilizing data from the National Health and Nutrition Examination Survey (NHANES).

## 2 Methods

### 2.1 Data source and study population

**Figure 1:**
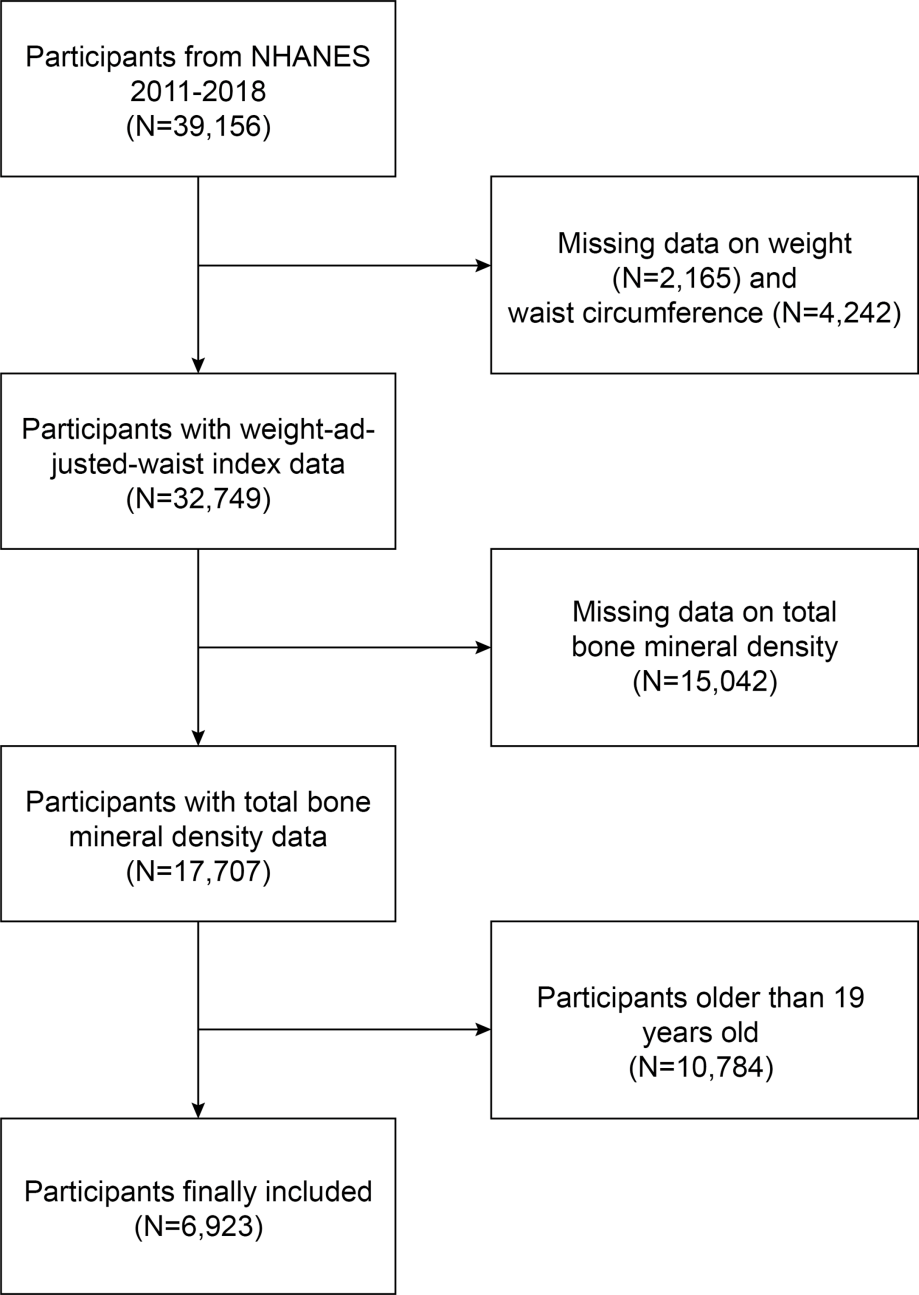
Baseline characteristics of participants.

NHANES is a comprehensive cross-sectional survey carried out in the United States to furnish impartial statistics regarding health concerns and population health issues (20–22). This study employed NHANES data from 2011 to 2018; out of 39,156 eligible participants, 2,165 lacked weight data, 4,242 lacked WC data, 15,042 lacked total BMD data, and 10,784 individuals aged over 19 were excluded. Ultimately, 6,923 adolescents were enrolled in the study (**Figure 1**).

### 2.2 Ethics statement

The NHANES protocol received approval from the National Research Ethics Committee on Health Statistics, and signed consent forms were obtained. Once anonymized, the NHANES data were made publicly available, enabling researchers to transform the data into a research-appropriate format. We adhered to research data usage guidelines, ensuring that data were employed solely for statistical analysis and that all experiments conformed to current standards and regulations. The authors did not access any information capable of identifying individual participants during or after data collection.

### 2.3 Study variables

In this study, the dependent variable is total BMD, with the expected independent variable being WWI. WWI (cm/√kg) is computed by dividing WC (cm) by the square root of body weight (kg). To guarantee data reliability, outliers were inspected as needed. Age, weight, and gender were employed to confirm data accuracy, and any erroneous data were eliminated. Total BMD was calculated using dual-energy X-ray absorptiometry results. Covariates included age, sex, race, ratio of family income to poverty (PIR), diabetes status, uric acid, albumin, alanine aminotransferase (ALT), direct high-density lipoprotein cholesterol (HDL-C), weight, alkaline phosphatase (ALP), 25OHD2 + 25OHD3, BMI, WC, aspartate aminotransferase (AST), blood urea nitrogen (BUN), glycated hemoglobin, creatinine, triglycerides, total cholesterol, total calcium, low-density lipoprotein cholesterol (LDL-C), phosphorus, and serum glucose. For additional information on confounding factors, visit http://www.cdc.gov/nchs/nhanes/.

### 2.4 Statistical analysis

Statistical evaluations in this investigation were executed using R (http://www.r-project.org) and EmpowerStats (http://www.empowerstats.com), adopting a significance threshold of p<0.05. As NHANES aims to generate data representative of the non-institutionalized civilian population within the United States, all estimates were computed utilizing sample weights in line with NCHS analytical guidelines (23, 24). Weighted multiple linear regression analysis was implemented to explore the linear relationship between WWI and total BMD, while smoothing curve fitting and threshold effects evaluation were applied to assess the non-linear association between WWI and total BMD. The study incorporated three models: Model 1 entailed no variable adjustments; Model 2 accounted for age, gender, and race; and Model 3 adjusted for all the covariates listed in **Table 1** except BMI, WC, and weight. Subgroup analyses were additionally performed.

**Table 1:** Basic characteristics of participants by weight-adjusted waist index quartile.

Characteristics	Weight-adjusted waist index (cm/√kg)				p-value
	Q1 (8.04–10.43) N=1,730	Q2 (10.43–11.00) N=1,731	Q3 (11.01–11.59) N=1,731	Q4 (11.59–15.21) N=1,731	
Age (years)	15.49 ± 2.34	14.10 ± 2.99	12.55 ± 3.33	11.18 ± 3.27	<0.0001
Sex (%)					<0.0001
Male	72.26	41.15	44.51	49.14	
Female	27.74	58.85	55.49	50.86	
Race/ethnicity (%)					<0.0001
Mexican American	8.96	13.58	17.67	23.22	
Other Hispanic	6.06	7.11	8.82	9.58	
Non-Hispanic White	52.94	56.95	53.01	50.97	
Non-Hispanic Black	21.89	12.72	10.32	7.64	
Other Race	10.16	9.63	10.18	8.59	
Diabetes (%)					<0.001
Yes	0.09	0.49	0.55	0.52	
No	99.76	99.22	99.01	98.46	
Borderline	0.15	0.29	0.44	1.02	
PIR	2.65 ± 1.64	2.63 ± 1.65	2.43 ± 1.62	2.13 ± 1.50	<0.0001
Albumin (g/dl)	4.52 ± 0.31	4.48 ± 0.31	4.41 ± 0.29	4.32 ± 0.28	<0.0001
ALT (U/L)	17.50 ± 13.04	17.77 ± 12.93	20.02 ± 16.20	23.78 ± 15.28	<0.0001
AST (U/L)	23.80 ± 11.89	22.84 ± 12.64	22.47 ± 8.36	23.57 ± 7.67	0.0304
ALP (IU/L)	143.50 ± 98.84	132.49 ± 94.96	132.09 ± 88.21	144.24 ± 91.42	0.0039
vitamin D (nmol/L)	65.77 ± 22.50	65.76 ± 23.58	63.12 ± 20.03	62.85 ± 18.67	<0.0001
BUN (mg/dl)	11.89 ± 3.46	11.07 ± 3.34	11.02 ± 3.69	10.48 ± 3.06	<0.0001
Total calcium (mg/dl)	9.62 ± 0.30	9.59 ± 0.29	9.58 ± 0.31	9.54 ± 0.32	<0.0001
Creatinine (mg/dl)	0.80 ± 0.16	0.70 ± 0.15	0.68 ± 0.15	0.64 ± 0.16	<0.0001
Serum glucose (mg/dl)	87.27 ± 10.27	88.87 ± 9.24	91.04 ± 15.17	89.90 ± 11.90	<0.0001
Phosphorus (mg/dl)	4.33 ± 0.67	4.31 ± 0.63	4.29 ± 0.69	4.29 ± 0.72	0.4666
Uric acid (mg/dl)	5.18 ± 1.13	4.82 ± 1.18	5.08 ± 1.31	5.36 ± 1.27	<0.0001
Glycohemoglobin (%)	5.21 ± 0.33	5.21 ± 0.36	5.23 ± 0.40	5.31 ± 0.47	<0.0001
Total cholesterol (mg/dl)	150.21 ± 26.44	157.59 ± 27.76	158.96 ± 29.01	161.99 ± 28.96	<0.0001
Triglyceride (mg/dl)	65.27 ± 41.15	77.71 ± 48.81	81.72 ± 51.48	100.09 ± 52.53	<0.0001
LDL-C (mg/dl)	81.83 ± 23.61	90.14 ± 25.02	91.42 ± 26.30	93.95 ± 26.99	<0.0001
Direct HDL-C (mg/dl)	54.15 ± 11.53	54.48 ± 12.25	52.56 ± 12.96	49.87 ± 12.27	<0.0001
Weight (kg)	61.41 ± 13.44	56.50 ± 18.35	54.88 ± 24.59	54.58 ± 26.89	<0.0001
BMI (kg/m^2^)	21.22 ± 3.48	21.71 ± 4.88	22.62 ± 6.64	24.30 ± 7.72	<0.0001
Waist circumference (cm)	73.55 ± 7.90	75.45 ± 12.17	77.74 ± 17.19	83.59 ± 20.55	<0.0001
WWI (cm/√kg)	9.45 ± 0.30	10.17 ± 0.16	10.75 ± 0.18	11.63 ± 0.44	<0.0001
Total BMD (g/cm^2^)	1.06 ± 0.13	0.97 ± 0.13	0.91 ± 0.15	0.85 ± 0.14	<0.0001

Mean ± SD for continuous variables: the p-value was calculated by the weighted linear regression model. (%) for categorical variables: the p-value was calculated by the weighted chi-square test.

Q, quartile; PIR, ratio of family income to poverty; BMI, body mass index; LDL-C, low-density lipoprotein cholesterol; BMD, bone mineral density; HDL-C, high-density lipoprotein cholesterol; AST, aspartate aminotransferase; ALT, alanine aminotransferase; ALP, alkaline phosphatase; BUN, blood urea nitrogen; vitamin D, 25OHD2 + 25OHD3.

## 3 Results

### 3.1 Baseline characteristics

After applying inclusion and exclusion criteria, 6,923 participants, with a mean age of 13.03 ± 3.45 years, were included in the study. The sample comprised 51.86% boys and 48.14% girls, along with 20.05% non-Hispanic white people, 24.38% non-Hispanic black people, 20.99% Mexican-American people, 10.63% other Hispanic people, and 16.94% individuals from other racial backgrounds. The average (SD) values of WWI and total BMD were 10.50 (0.86) cm/√kg and 0.94 (0.16) g/cm^2^, respectively. **Table 1** outlines the clinical characteristics of the participants, with columns displaying stratified groups based on WWI quartiles. Relative to the bottom quartile, those in the top WWI quartile were more likely to be female and younger, have a higher proportion of Mexican Americans and other Hispanics, exhibit a higher prevalence of diabetes, and show increased levels of ALT, ALP, serum glucose, uric acid, glycohemoglobin, total cholesterol, triglyceride, LDL-C, BMI, and WC. Conversely, they demonstrated lower levels of PIR, albumin, AST, 25OHD2 + 25OHD3, BUN, total calcium, creatinine, direct HDL-C, weight, and total BMD (p < 0.05) (**Table 1**).

### 3.2 Association between WWI and total BMD

**Table 2** presents the connection between WWI and total BMD. All models exhibited an inverse association between WWI and total BMD. Upon adjusting for all confounding variables, a one-unit increment in WWI was significantly linked to a 0.03 g/cm^2^ reduction in total BMD (Model 3: β = −0.03, 95% CI: −0.03, −0.03). This correlation persisted as statistically significant when categorizing WWI into quartiles: a one-unit rise in WWI corresponded to a 0.06-unit larger decrease in total BMD for cipants in the top WWI quartile compared to those in the bottom WWI quartile (β = -0.06, 95% CI: −0.06, −0.05; p for trend < 0.001).

**Table 2:** Association between weight-adjusted waist index (cm/√kg) and total bone mineral density (g/cm^2^).

Exposure	Model 1 [β (95% CI)]	Model 2 [β (95% CI)]	Model 3 [β (95% CI)]
WWI (continuous)	−0.09 (−0.10, −0.09)	−0.02 (−0.03, −0.02)	−0.03 (−0.03, −0.03)
WWI (quartile)			
Quartile 1	Reference	Reference	Reference
Quartile 2	−0.09 (−0.10, −0.08)	−0.03 (−0.04, −0.02)	−0.03 (−0.03, −0.02)
Quartile 3	−0.15 (−0.16, −0.14)	−0.04 (−0.05, −0.04)	−0.05 (−0.05, −0.04)
Quartile 4	−0.21 (−0.22, −0.20)	−0.05 (−0.05, −0.04)	−0.06 (−0.06, −0.05)
*p* for trend	<0.001	<0.001	<0.001

Model 1: no covariates were adjusted. Model 2: age, gender, and race were adjusted. Model 3: age, gender, race, diabetes, PIR, albumin, ALT, AST, ALP, vitamin D, BUN, total calcium, creatinine, serum glucose, phosphorus, uric acid, glycohemoglobin, total cholesterol, triglyceride, LDL-C, and direct HDL-C were adjusted.

PIR, ratio of family income to poverty; LDL-C, low-density lipoproteincholesterol; HDL-C, high-density lipoprotein cholesterol; AST, aspartate aminotransferase; ALT, alanine aminotransferase; ALP, alkaline phosphatase; BUN, blood urea nitrogen; vitamin D, 25OHD2 + 25OHD3.

### 3.3 Subgroup analysis

A subgroup analysis was conducted to evaluate the stability of the relationship between WWI and total BMD across different demographic contexts. The results indicated that the influence of factors other than age stratification on the relationship between WWI and total BMD was not significant. As illustrated in **Table 3**, the inverse association between WWI and total BMD was not significantly affected by any of the remaining strata, including gender, ethnicity, diabetes status, and the ratio of family income to poverty, except for the age stratum (p > 0.05 for all interactions). However, in the age-stratified subgroup analyses, the absolute effect values for 8–11-year-olds were significantly smaller than those for 12–19-year-olds. This implies that each unit increase in WWI for 8–11-year-olds was associated with a 0.01 cm/√kg decrease in total BMD, while each unit increase in WWI for 12–19-year-olds corresponded to a 0.03 cm/√kg decrease in total BMD. Furthermore, the results of the WWI quartile subgroup analysis demonstrated a dose–response relationship between WWI and total BMD (**Table 3**).

**Table 3:** Association between weight-adjusted waist index (cm/√kg) and total bone mineral density (g/cm^2^).

	β (95% CI)	*p* for interaction
Stratified by gender		0.3371
Male	1.20 (1.07, 1.35)	
Female	1.12 (1.03, 1.21)	
Stratified by race		0.6982
Mexican American	−0.03 (−0.04, −0.02)	
Other Hispanic	−0.03 (−0.04, −0.02)	
Non-Hispanic White	−0.03 (−0.03, −0.03)	
Non-Hispanic Black	−0.02 (−0.03, −0.02)	
Other Race	−0.03 (−0.04, −0.02)	
Stratified by age		<0.0001
8–9 years old	−0.01 (−0.02, −0.00)	
10–11 years old	−0.02 (−0.02, −0.01)	
12–13 years old	−0.03 (−0.03, −0.02)	
14–15 years old	−0.02 (−0.02, −0.01)	
16–17 years old	−0.02 (−0.02, −0.01)	
18–19 years old	−0.03 (−0.04, −0.02)	
Stratified by PIR		0.2997
<1.3	−0.03 (−0.03, −0.02)	
1.3–3.5	−0.03 (−0.03, −0.02)	
>3.5	−0.03 (−0.04, −0.03)	
Stratified by diabetes		0.9309
Yes	−0.01 (−0.11, 0.08)	
No	−0.03 (−0.03, −0.03)	
Borderline	−0.03 (−0.11, 0.04)	

In subgroup analyses stratified by sex, race, age, and diabetes status. The model adjusted for covariates such as age, sex, race, diabetes, PIR, albumin, ALT, AST, ALP, vitamin D, BUN, total calcium, creatinine, serum glucose, phosphorus, uric acid, glycohemoglobin, total cholesterol, triglycerides, LDL-C, and direct HDL-C, but the model did not adjust for the stratification variables themselves.

PIR, ratio of family income to poverty; LDL-C, low-density lipoproteincholesterol; HDL-C, high-density lipoprotein cholesterol; AST, aspartate aminotransferase; ALT, alanine aminotransferase; ALP, alkaline phosphatase; BUN, blood urea nitrogen; vitamin D, 25OHD2 + 25OHD3.

### 3.4 Non-linearity and saturation effect analysis between WWI and total BMD

**Figure 2:**
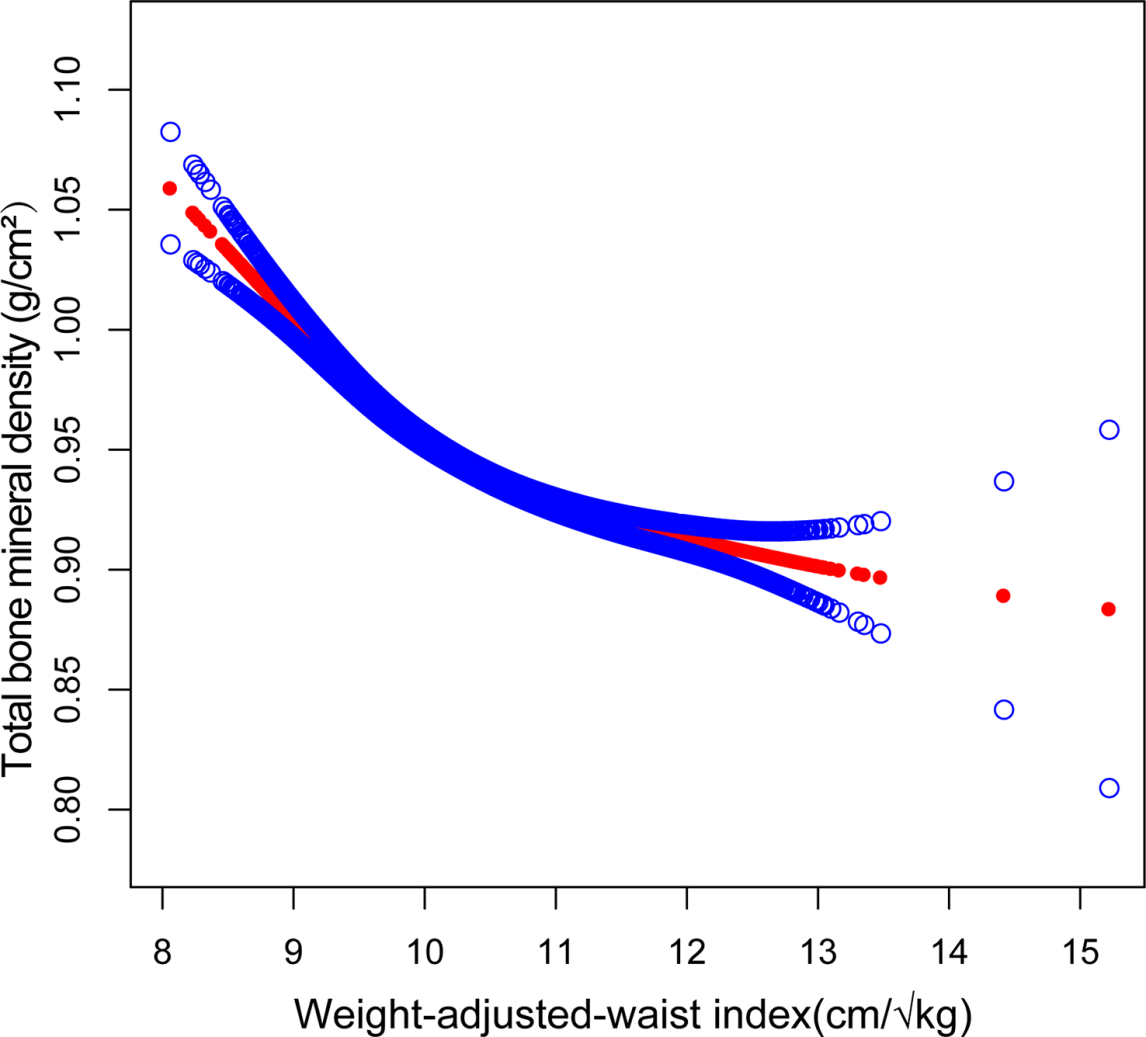
Association between WWI and total bone mineral density (The solid red line represents the smooth curve fit between variables. Blue bands represent the 95% confidence interval from the fit).

**Figure 3:**
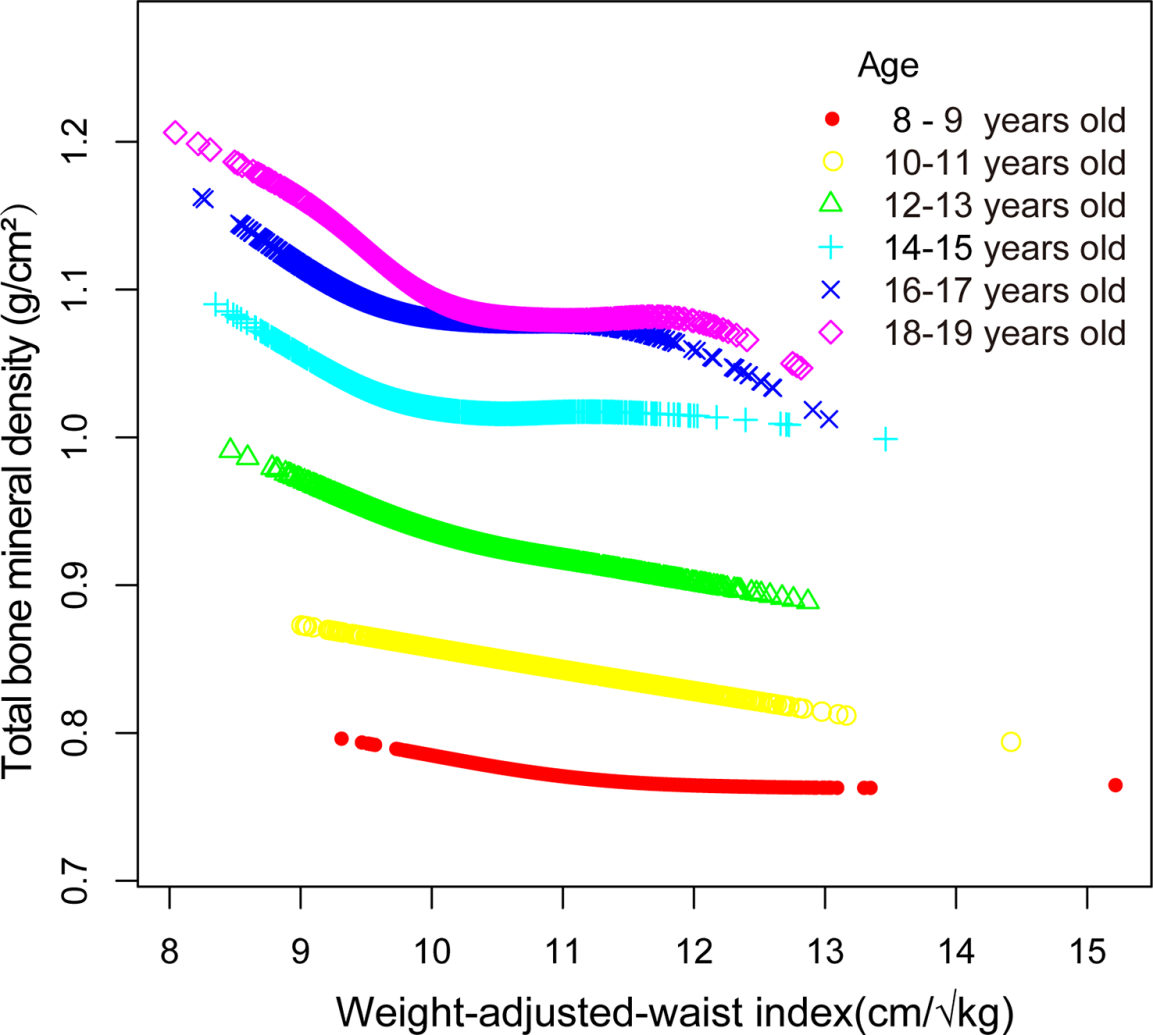
Association between WWI and total bone mineral density stratified by age.

Smooth curve fitting was implemented to delineate the non-linear association and saturation phenomenon between WWI and total BMD (**Figure 2**). The results uncovered that the saturation effect value for the WWI–total BMD relationship was 9.88 cm/√kg for all participants (**Table 4**). When WWI was below 9.88 cm/√kg, the effect value registered at −0.06; on the other hand, when WWI surpassed 9.88 cm/√kg, the effect value transitioned to −0.02. All participants were segregated into three groups with 2-year age intervals. Smooth curves and saturation effect evaluation were applied to ascertain WWI saturation values for total BMD within each age bracket (**Table 4**; **Figure 3**).

**Table 4:** Saturation effect analysis of WWI (cm/√kg) on total BMD (g/cm^2^).

Total bone mineral density	Model: saturation effect analysis [β (95% CI) P value]
WWI turning point (K)	9.88
<K, effect1	−0.06(−0.07, −0.05) <0.0001
>K, effect2	−0.02(−0.03, −0.02) <0.0001
Log-likelihood ratio	<0.001
Subgroup analysis stratified by age	
WWI turning point for 8–9 years old (K)	10.55
<K, effect1	−0.04 (−0.07, −0.02) 0.0003
>K, effect2	−0.01 (−0.01, −0.00) 0.0477
Log-likelihood ratio	0.006
WWI turning point for 10–11 years old (K)	11.3
<K, effect1	−0.02 (−0.03, −0.02) <0.0001
>K, effect2	−0.00 (−0.02, 0.01) 0.5770
Log-likelihood ratio	0.057
WWI turning point for 12–13 years old (K)	9.91
<K, effect1	−0.06 (−0.09, −0.04) <0.0001
>K, effect2	−0.02 (−0.03, −0.01) 0.0004
Log-likelihood ratio	0.002
WWI turning point for 14–15 years old (K)	9.87
<K, effect1	−0.06 (−0.08, −0.03) <0.0001
>K, effect2	0.00 (−0.01, 0.02) 0.4910
Log-likelihood ratio	<0.001
WWI turning point for 16–17 years old (K)	9.51
<K, effect1	−0.07 (−0.10, −0.03) <0.0001
>K, effect2	−0.01 (−0.02, 0.00) 0.1501
Log-likelihood ratio	0.001
WWI turning point for 18–19 years old (K)	10.00
<K, effect1	−0.07 (−0.09, −0.04) <0.0001
>K, effect2	0.00 (−0.01, 0.01) 0.9354
Log-likelihood ratio	<0.001

Age, gender, race, diabetes status, albumin, ALT, AST, ALP, BMI, vitamin D, BUN, total cholesterol, total calcium, creatinine, phosphorus, glycohemoglobin, triglyceride, LDL-C, direct HDL-C, uric acid, serum glucose, refrigerated serum, and PIR were adjusted.

PIR, ratio of family income to poverty; LDL-C, low-density lipoproteincholesterol; HDL-C, high-density lipoprotein cholesterol; AST, aspartate aminotransferase; ALT, alanine aminotransferase; ALP, alkaline phosphatase; BUN, blood urea nitrogen; vitamin D, 25OHD2 + 25OHD3.

## 4 Discussion

Our cross-sectional investigation uncovered a notable negative correlation between WWI and total BMD among adolescents. Subgroup analyses and interaction evaluations revealed that this negative correlation remained consistent across all subcategories, with the exception of age, encompassing gender, ethnicity, and diabetes status subgroups. Notably, we discerned an L-shaped association between WWI and total BMD, exhibiting an inflection point at 9.98 cm/√kg.

To the best of our knowledge, this constitutes the first cross-sectional investigation exploring the relationship between WWI and total BMD. Globally, recognized standards for defining obesity include BMI and WC (14, 25–27). Numerous studies have reported a positive correlation between BMI, WC, and BMD (28, 29). Leonard, et al. concluded that childhood and adolescent obesity is linked to increased BMD by analyzing data from 182 children and adolescents aged 4–20 years, gathered from the Nutrition and Growth Laboratory at the Children’s Hospital of Philadelphia, and categorizing them into obese and non-obese groups based on BMI and WC (30). However, due to the limited sample size in this study, additional research with a larger sample is warranted. Ouyang et al. incorporated data from 6,143 adolescents aged 8–19 from the 2011–2020 NHANES and utilized smooth curve fitting and threshold effect analysis for statistical evaluation. The smooth curve fitting results revealed a significant positive association between BMI and BMD. Threshold effect analysis determined that maintaining BMI at saturation (21.5kg/m^2^) could minimize other adverse consequences and optimize BMD (31). Wang et al. evaluated the association between BMI, WC, and BMD by incorporating data from 4,056 US adolescents aged 8–19 years from the 2011–2018 NHANES. They concluded that both BMI and WC were positively associated with total BMD and that a saturation effect of BMD could be achieved by maintaining BMI at 22 kg/m^2^ and WC at 70.5 cm (32). However, as research has progressed, some scholars have discovered an obesity paradox when using BMI and WC as obesity measures. The obesity paradox suggests that obesity does not necessarily shorten patients’ expected survival time and that overweight individuals may have a slightly lower risk of death compared to those with normal weight and may even exhibit beneficial effects in some cases. The existence of the obesity paradox raises questions about the validity of BMI and WC as obesity measures among researchers (33–36). Consequently, identifying an obesity index that eliminates the obesity paradox is essential; WWI is a recently devised anthropometric index that has gained recognition as a reliable obesity measure alongside BMI and WC, attributed to its straightforward calculation and capacity to discern between lean and fat masses (37, 38). Contemporary research has substantiated WWI’s ability to differentiate muscle mass from fat mass, and its application has extended to various fields, including cardiovascular disease and obesity (39). Numerous studies have demonstrated that WWI is a distinct anthropometric index positively associated with heart failure incidence and mortality rates (40, 41). Remarkably, the obesity paradox observed in the relationship between BMI and mortality is absent in the association between WWI and mortality (42). Moreover, some researchers contend that the obesity paradox might not exist at all, potentially due to BMI’s limitations in differentiating between muscle mass and fat mass (43, 44). Our findings diverge from prior studies. While earlier research has indicated positive correlations between BMI, WC, and total BMD, our investigation identified a negative association between WWI and total BMD.

At present, the potential mechanisms underpinning the observed negative association between WWI and total BMD are not well elucidated. Several mechanisms might be involved (32). First, excessive body fat accumulation and elevated obesity levels result in increased static mechanical compliance, imposing various static mechanical stresses on bones and inducing alterations in bone structure (45–47). Subsequently, research has indicated that obesity augments the population of adipocytes within the bone marrow while concurrently modifying their metabolic function. The bone marrow houses bone mesenchymal stem cells (BMSCs), which possess the capacity to differentiate into both osteoblasts and adipocytes (48, 49). The presence of obesity has been demonstrated to promote the differentiation of BMSCs towards adipocytes, culminating in a heightened presence of adipocytes within the bone marrow and a concomitant reduction in the osteoblast population (50). Excessive fat cell accumulation within the bone marrow results in an imbalance in osteocyte activity and diminished bone turnover, predisposing individuals to surgical intervention at a younger age (51). Moreover, obesity is associated with an increased susceptibility to inflammation (52). The expansion of adipocytes in the bone marrow microenvironment not only stimulates osteoclastogenesis and activation but also restricts osteoprotegerin secretion, inhibits osteoblast differentiation, and expedites the release of inflammatory and immunomodulatory factors that facilitate osteoclast formation (53, 54).

The conclusions drawn from this study warrant careful consideration, given several constraints. Primarily, the study’s cross-sectional framework hinders the establishment of a causal link between WWI and total BMD. Additionally, database restrictions precluded the collection of information regarding lifestyle, dietary habits, bone metabolism parameters, and calcium and vitamin D intake for all participants, which may have impacted total BMD. Lastly, the unavailability of youth fracture data due to database limitations rendered it infeasible to ascertain whether the fracture incidence was elevated in young individuals with higher WWI compared to the general population. Notwithstanding the aforementioned constraints, the merits of this investigation warrant recognition. Primarily, employing a nationally representative cohort ensured that the results encapsulated the heterogeneity of the youth demographic across the United States. Additionally, the substantial sample size facilitated subgroup analyses by segregating participants aged 8–19 into distinct age categories.

## 5 Conclusion

This investigation’s outcomes reveal an inverse association and saturation phenomenon between WWI and total BMD among American adolescents. The implications suggest that maintaining WWI within the optimal range could be vital for effectively managing bone metabolic health during this developmental stage. Nonetheless, to corroborate these conclusions, additional longitudinal research with more extensive cohorts is required.

## Data availability statement

Publicly available datasets were analyzed in this study. This data can be found here: https://www.cdc.gov/nchs/nhanes/index.htm.

## Ethics statement

The studies involving human participants were reviewed and approved by The National Research Ethics Committee. Written informed consent to participate in this study was provided by the participants’ legal guardian/next of kin.

## Author contributions

XW and LX designed the research. XW, SY, and GH collected and analyzed the data. XW drafted the manuscript. LX revised the manuscript. All authors contributed to the article and approved the submitted version.
